# A Proposed Parameter-Based Planning Framework for Inferior Alveolar Nerve Repositioning

**DOI:** 10.3390/dj14090561

**Published:** 2026-09-03

**Authors:** Fares Kablan, Asaf Zigron, Idan Redenski, Shadi Daoud, Amjad Shhadeh, Adeeb Zoabi, Samer Srouji

**Affiliations:** 1 Department of Oral and Maxillofacial Surgery, Galilee College of Dental Sciences, Galilee Medical Center, Nahariya 2210001, Israel; 2 The Azrieli Faculty of Medicine, Bar-Ilan University, Safed 1311502, Israel

**Keywords:** inferior alveolar nerve repositioning, nerve lateralization, nerve transposition, atrophic posterior mandible, dental implants

## Abstract

**Background**: Rehabilitation of the severely atrophic posterior mandible remains challenging when the residual bone height above the inferior alveolar nerve (IAN) is insufficient for conventional implant placement. In such cases, IAN lateralization or transposition may facilitate placement of longer implants and support fixed prosthetic rehabilitation. However, these procedures require careful case selection because of their technical complexity and the risk of neurosensory complications. **Objective**: This narrative review presents a literature-informed, expert-derived, parameter-based clinical decision framework for preoperative planning of IAN lateralization and transposition in the severely atrophic posterior mandible. **Methods**: A focused appraisal of the literature was performed to identify anatomical, prosthodontic, and surgical parameters relevant to IAN repositioning. Parameters were selected according to their recurrent relevance to surgical feasibility, implant positioning, procedural safety, and restorative planning, and were integrated with the authors’ clinical experience to develop a structured decision-making framework. No formal risk-of-bias or certainty-of-evidence assessment was performed. Each parameter was assigned an evidence-source category and a threshold status to make its evidentiary basis explicit. **Results**: The proposed framework organizes treatment planning into three domains: bone-related, prosthodontic, and surgical considerations. Key parameters include residual bone height above the IAN canal, global mandibular height, ridge width, bone density, crown-to-implant ratio, intermaxillary space, ridge angulation, buccal cortical plate thickness, and the three-dimensional course of the IAN. Additional parameters include interforaminal dentition, implant trajectory, canal diameter, bifid canal anatomy, and ridge contour. Collectively, these variables are integrated into a stepwise decision pathway to help determine whether IAN repositioning is appropriate, whether lateralization or transposition is preferable, and when alternative approaches, such as short implants, ridge splitting, or vertical augmentation, may be more suitable. The framework links restorative feasibility and surgical risk to the choice of technique within a single sequential pathway, and reports the evidence basis of each decision point. **Conclusions**: The proposed framework should not be regarded as a validated clinical decision tool; prospective clinical validation and consensus-based refinement are required before routine clinical implementation. The framework is intended to structure and guide multidisciplinary assessment and discussion of cases; it should not independently determine treatment selection or replace individualized clinical judgment.

## 1. Introduction

Rehabilitation of the atrophic posterior mandible remains one of the most complex and technique-sensitive procedures in implant dentistry. Progressive alveolar resorption following tooth loss leads to a significant reduction in vertical ridge height, often leaving minimal bone height above the inferior alveolar nerve (IAN). This condition restricts the placement of endosseous implants and increases the risk of direct nerve injury and postoperative neurosensory disturbances. When the residual bone height becomes critically insufficient, IAN lateralization or transposition may provide a viable surgical option to gain adequate vertical space and permit the placement of longer, biomechanically stable implants in a single stage [1,2,3]. The concept of IAN repositioning was first described by Alling in 1977 [4] and was later modified by Jensen and Nock in 1987 [5], who introduced the use of endosseous implants in conjunction with IAN transposition for rehabilitation of the atrophic posterior mandible.

The essential distinction between these two approaches lies in the management of the incisive branch. Lateralization entails exposure and temporary retraction of the IAN while maintaining its continuity and preserving the vitality of the anterior teeth, whereas transposition requires release of the mental nerve and deliberate sectioning of the incisive branch to permit posterior repositioning of the entire neurovascular bundle after anterior release at the mental foramen. Both procedures can achieve stable implant anchorage in severely atrophic mandibles; however, the literature reports a higher incidence of persistent postoperative neurosensory disturbance and loss of anterior tooth vitality following transposition than following lateralization [1,2,3,6].

Indications for IAN repositioning are limited to cases of severe mandibular atrophy, in which even short implants cannot provide sufficient anchorage without risking nerve trauma [7,8,9]. The technique is particularly recommended when fixed prosthetic rehabilitation is required and when vertical augmentation methods are contraindicated or are expected to provide inadequate or unstable volume [8]. Advances in preoperative imaging, particularly cone-beam computed tomography (CBCT), have improved case selection by providing precise three-dimensional mapping of the mandibular canal and enabling virtual simulation of implant trajectories [10].

Despite its advantages, IAN repositioning should not be regarded as the sole treatment option for mandibular atrophy. Short implants offer a minimally invasive alternative when ≥6 mm of bone is available, yet their biomechanical performance in high-load posterior regions has been questioned on the basis of unfavorable crown-to-implant ratios [11,12]. Ridge splitting addresses horizontal ridge deficiency but not the vertical bone loss that characterizes severe atrophy [13]. Vertical augmentation methods can restore ridge height, but they require staged procedures, are associated with donor-site morbidity, and may be affected by unpredictable resorption [14,15]. When these alternatives are contraindicated or insufficient, IAN repositioning remains the most direct approach to fixed implant-supported rehabilitation in a single stage.

**Figure 1:**
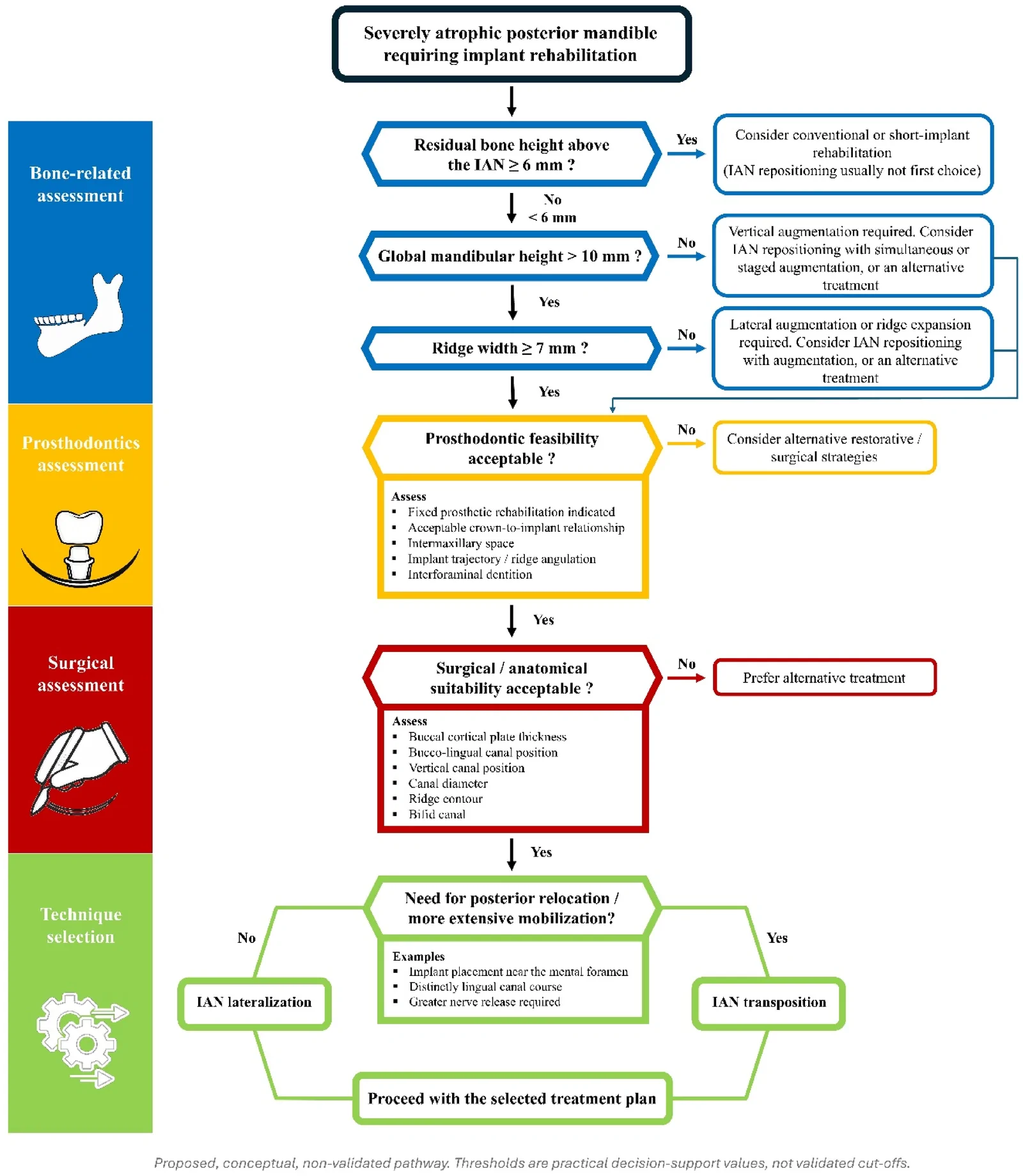
Proposed stepwise clinical decision flowchart for inferior alveolar nerve repositioning. The pathway begins with assessment of residual bone height above the inferior alveolar nerve (IAN) and sequentially integrates global mandibular height, ridge width, prosthodontic feasibility, and surgical/anatomical factors. The flowchart guides selection among alternative treatment options, IAN repositioning with or without adjunctive augmentation, and lateralization or transposition. Thresholds shown in the flowchart are intended as practical decision-support values derived from prior classifications, published clinical studies, and established implant-planning principles, and should not be interpreted as universally validated cutoff values. The pathway is proposed, conceptual, and requires individualized clinical consideration. The evidence category and threshold status of each decision node are reported in Table 1.

Although the body of literature on IAN repositioning techniques and outcomes continues to grow, a structured, parameter-based framework for preoperative decision-making remains lacking. Clinicians often rely on personal experience or informal criteria when selecting candidates, leading to inconsistent case selection and variable outcomes. To address this gap, the current work introduces a literature-informed, expert-derived, parameter-based clinical decision framework for preoperative evaluation in IAN repositioning. The framework organizes key determinants into bone-related, prosthodontic, and surgical domains and is intended to support a more structured and consistent treatment-planning approach. To translate these considerations into a clinically usable format, a stepwise clinical decision flowchart is proposed (Figure 1). This flowchart sequentially integrates the key bone-related, prosthodontic, and surgical parameters to help clinicians determine whether IAN repositioning may be indicated, whether adjunctive augmentation or alternative treatment should be considered, and whether lateralization or transposition may be the more appropriate surgical option.

**Table 1:** Summary of parameters included in the proposed clinical decision framework for inferior alveolar nerve repositioning.

Domain	Parameter	Clinical Implication	Suggested Threshold/Decision Value	Main Supporting Evidence
Bone-related	Residual bone height above the IAN	Primary indication for considering IAN repositioning versus conventional or short-implant placement	<6 mm favors consideration of IAN lateralization/transposition; ≥6 mm may allow short implants in selected cases	Clinical studies and reviews on IAN repositioning and short implants [1,2,3,12], Category A (direct IAN repositioning evidence); threshold status: pragmatic.
Bone-related	Global mandibular height	Determines whether repositioning alone can provide adequate implant anchorage or whether augmentation is required	>10 mm may allow repositioning alone; ≤10 mm may require simultaneous or staged vertical augmentation	Kablan classification and augmentation literature [13,14,15,16], Category D (classification-derived); threshold status: pragmatic.
Bone-related	Ridge width	Determines feasibility of standard-diameter implant placement and need for lateral augmentation	Approximately ≥7 mm crestal width; inadequate width favors lateral augmentation/expansion	Implant-planning principles and ridge augmentation literature [17,18,19], Category B (extrapolated from general implant literature); threshold status: extrapolated.
Bone-related	Basal/apical bone width beneath the canal	Influences primary stability after nerve mobilization	Commonly >6 mm beneath the canal when apical anchorage is required	Classification-based and clinical planning recommendations [16,20], Category D (classification-derived); threshold status: pragmatic.
Bone-related	Bone quality	Influences osteotomy technique, thermal/mechanical risk, and primary implant stability	D1 bone may favor piezosurgery; D3–D4 may require longer implants and bicortical engagement	Surgical technique reviews and piezosurgery studies [16,21,22,23], Category A/C (technique and in vitro evidence); threshold status: pragmatic.
Prosthodontic	Interforaminal dentition	Favors preservation of restorable anterior/premolar teeth and may influence choice of IAN repositioning technique	Presence of restorable interforaminal teeth	Prosthodontic rationale and clinical experience in partially edentulous mandibles [6,24], Category E (expert judgment); threshold status: pragmatic.
Prosthodontic	Crown-to-implant ratio	Influences biomechanical planning, splinting, and choice between short and longer implants	No universal cutoff; unfavorable ratios may favor longer implants or splinted restorations	Biomechanical and systematic-review evidence [24,25,26,27], Category B (extrapolated from general implant literature); no threshold proposed—evidence conflicting, requires individualized assessment.
Prosthodontic	Intermaxillary space	Determines prosthetic design and whether vertical augmentation is prosthetically favorable	Excessive space may require hybrid/overdenture design; reduced space may make vertical augmentation less favorable	Prosthodontic restorative-space literature [28], Category B (extrapolated from general prosthodontic literature); threshold status: extrapolated.
Prosthodontic	Ridge/implant angulation	Determines feasibility of prosthetically driven implant placement	No fixed cutoff; unfavorable angulation may require guided planning, augmentation, or alternative strategy	Guided implant surgery and navigation literature [23,29,30], Category B (extrapolated from general implant literature); no threshold proposed.
Surgical	Buccal cortical plate thickness	Influences lateral window design, feasibility of replacing the bony window, and risk of fragmentation	Thick cortex may permit replaceable window; thin/porous cortex may require membrane/grafting	Surgical technique literature [16,23], Category A (direct IAN repositioning evidence); threshold status: pragmatic.
Surgical	IAN bucco-lingual position	Determines depth of access, degree of nerve mobilization, and traction risk	Lingual canal position may require wider exposure and may favor transposition in selected cases	CBCT and surgical access literature [8,10,23], Category C (anatomical/imaging evidence); threshold status: pragmatic.
Surgical	IAN vertical position/anterior loop	Determines osteotomy height, extent of exposure, and access strategy	High/superficial canal requires wider exposure; extremely superficial canal may allow superior access	CBCT-based planning and superioralization technique [10,31], Category C/E (imaging evidence and authors’ described technique); threshold status: pragmatic.
Surgical	Mandibular canal diameter	Influences safe lateral displacement and traction risk	No validated cutoff; larger canals may limit safe mobilization	Anatomical studies and nerve injury literature [32,33], Category C (anatomical evidence); no validated cutoff exists.
Surgical	Bifid mandibular canal	Increases anatomical complexity and risk of incomplete mobilization or iatrogenic nerve injury	Presence of bifid canal is a relative contraindication; alternatives should be considered	CBCT, anatomical, and clinical relevance studies [34,35,36,37], Category C (anatomical/imaging evidence); threshold status: pragmatic.
Surgical	Ridge contour	Influences guide seating, implant positioning, flap closure, hygiene, and need for augmentation/alveoloplasty	Concave or knife-edge ridges may require GBR, block grafting, or conservative alveoloplasty	Edentulous ridge classification and augmentation literature [38,39,40], Category B/D (general literature and classification-derived); threshold status: pragmatic.

## 2. Framework Development

This article was developed as a narrative, clinically derived decision framework rather than as a formal study protocol or systematic review. The parameters were identified from a focused appraisal of the published literature, and the decision structure and thresholds reflect the authors’ interpretation of that literature combined with their cumulative clinical experience. A focused literature appraisal was performed to identify parameters relevant to IAN lateralization and transposition for implant rehabilitation of the atrophic posterior mandible. Relevant publications were identified through targeted searches of PubMed/MEDLINE and Google Scholar, supplemented by manual screening of reference lists from key reviews, clinical studies, anatomical studies, technical reports, and existing classifications. The appraisal was conducted iteratively by the authors. Searches were performed in English, and no publication-date restriction was applied; the cited literature accordingly spans the period from the original description of the technique in 1977 to present day. Of the cited publications, seven were systematic reviews or meta-analyses, eleven were clinical outcome studies of IAN lateralization or transposition, seven were anatomical or CBCT-based imaging studies, seven were surgical technique reports or in vitro studies, two were classification papers, and fifteen were drawn from the general implant and prosthodontic literature in which nerve repositioning was not performed. Recommendations resting on this category are extrapolated from studies in which nerve repositioning was not performed and are identified accordingly in Table 1. Search concepts included IAN lateralization, IAN transposition, IAN repositioning, atrophic posterior mandible, dental implants, mandibular canal, CBCT, short implants, ridge augmentation, and neurosensory disturbance.

Parameters were selected when they were relevant to at least one of the following: indication for IAN repositioning; choice between lateralization, transposition, augmentation, or alternative treatment; implant positioning or prosthetic feasibility; or risk of neurosensory or surgical complications. A parameter was retained only if it was measurable or directly observable on preoperative CBCT or clinical examination, and capable of altering at least one branch of the decision pathway. Parameters relating to intraoperative or postoperative management rather than preoperative planning were excluded. Parameter selection was performed by four of the authors (F.K., S.S., A.Zo., A.Zi.), who first reviewed the retrieved literature independently and then compared their proposed parameter sets. Disagreements regarding inclusion of a parameter or the value of a proposed threshold were resolved by decision of the two senior authors (S.S. and F.K.). Evidence was interpreted according to source type and clinical applicability. Systematic reviews and clinical outcome studies were prioritized when available, whereas anatomical, CBCT-based, technical, and classification-based studies were used when direct clinical outcome data were limited. When direct evidence was insufficient, expert clinical judgment was incorporated to formulate pragmatic decision-support recommendations. Expert judgment was applied only to two operations: proposing a working value where no published value existed, and ordering the parameters into a sequence. It was not used to override published clinical outcome data. Where sources conflicted, the discrepancy was retained rather than resolved by selecting a single value, and no binary threshold was proposed. Thus, such parameters are presented as requiring individualized assessment.

Since the available evidence is heterogeneous, the proposed thresholds should be interpreted as practical decision-support values rather than universally validated standards. No pooled quantitative estimates were generated, as this article was not designed as a systematic review or meta-analysis. To improve transparency, Table 1 summarizes the principal parameters included in the framework, their clinical implications, suggested decision thresholds when applicable, and the main supporting references. To make the evidentiary basis of each parameter explicit and traceable, these are assigned to one of five source categories: (A) direct clinical evidence from IAN repositioning studies; (B) extrapolation from the general implant or prosthodontic literature, in which the underlying studies did not involve nerve repositioning; (C) anatomical or CBCT-based imaging evidence; (D) derivation from a previously published classification, and (E) expert judgment. Each threshold is additionally labelled by status either as validated, where it has been tested against clinical outcomes in the specific context of IAN repositioning; extrapolated, where a published value exists but was established in a different clinical context; or pragmatic, where it represents a working value proposed by the authors to structure the decision pathway.

## 3. Key Factors in Treatment Planning

Based on the framework-development process described above, the selected parameters are summarized in Table 1 and are presented below according to three interrelated domains that reflect the multidisciplinary nature of treatment planning:

(1) Bone-related considerations, addressing mandibular bone quality, quantity, and morphology.
(2) Prosthodontic considerations, focusing on biomechanical demands, implant positioning, angulation, and occlusal load distribution.
(3) Surgical considerations, encompassing anatomical constraints, surgical access, and intraoperative safety.

A structured and sequential evaluation of these domains forms the basis of the proposed clinical decision framework. The stepwise application of this framework is illustrated in Figure 1. The decision pathway begins with assessment of residual bone height above the IAN and then proceeds through evaluation of mandibular height, ridge width, prosthodontic feasibility, and surgical/anatomical factors. At each stage, the flowchart identifies whether IAN repositioning is appropriate, whether adjunctive augmentation is required, whether an alternative treatment should be preferred, and when lateralization or transposition is the more suitable technique.

## 4. Bone-Related Considerations

**1.**  **Available bone height above the IAN**

**Figure 2:**
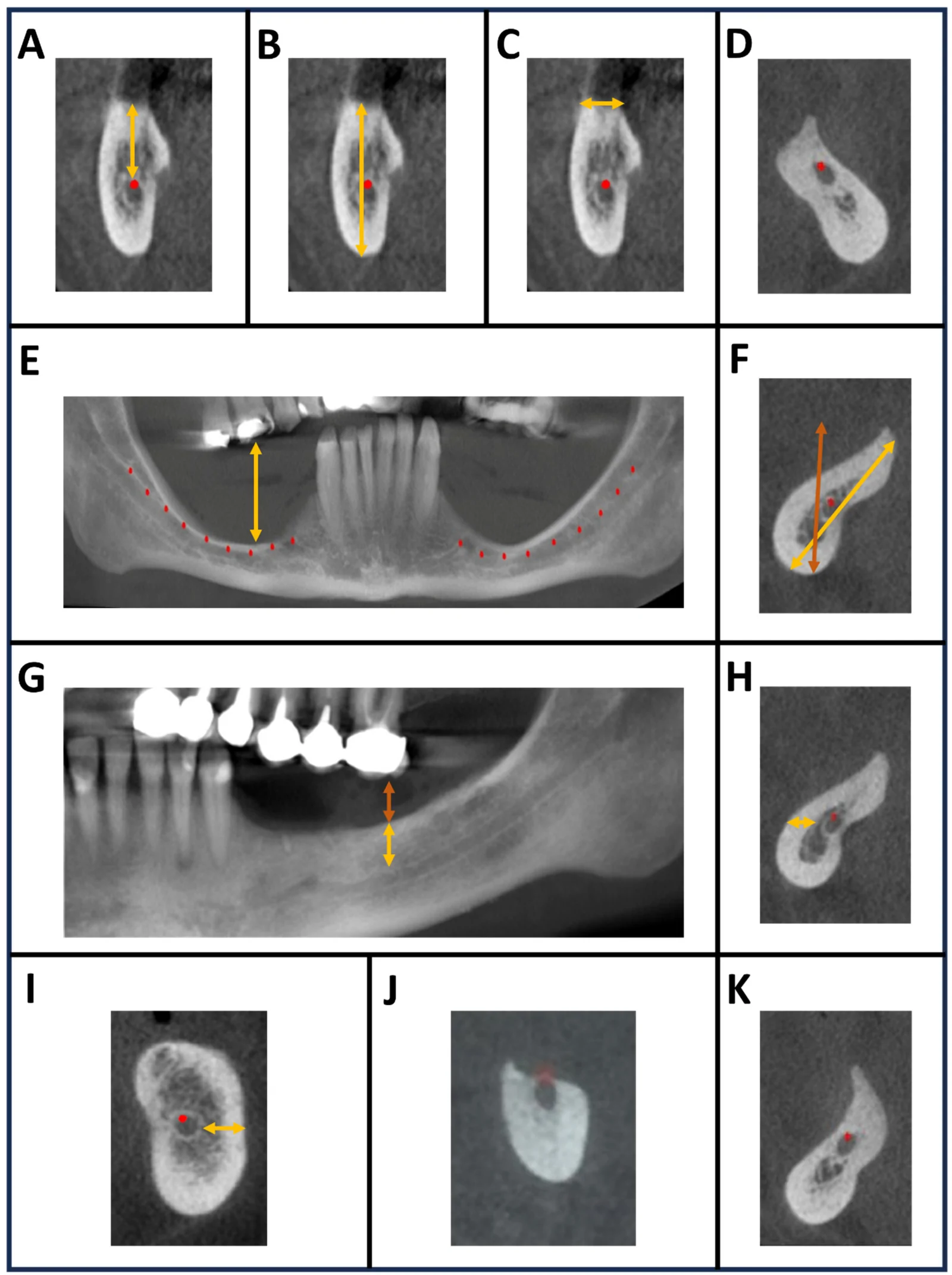
**Key radiographic parameters guiding preoperative planning for inferior alveolar nerve repositioning.** (**A**) Crest-to-canal height; (**B**) global mandibular height; (**C**) buccolingual crestal width; (**D**) bone density evaluation (D1); (**E**) excessive intermaxillary space; (**F**) ridge inclination relative to the desired implant axis; (**G**) reduced intermaxillary space; (**H**) buccal cortical plate thickness relevant to lateral window design; (**I**) lingual position of the mandibular canal; (**J**) high vertical course of the canal; (**K**) knife-edge ridge requiring alveoloplasty.

Residual vertical bone height from the alveolar crest to the superior border of the IAN canal is a key factor in determining whether nerve lateralization or transposition should be considered (Figure 2A). When the residual bone height is <6 mm, conventional short implant placement is often not feasible, and the resulting crown-to-implant ratio may become biomechanically unfavorable [1,2]. This threshold therefore serves as a practical entry point for considering nerve lateralization or transposition to achieve adequate implant length and primary stability [1,3].

**2.**  **Global mandibular height**

Global mandibular height refers to the total vertical dimension of the mandible measured from the alveolar crest to the inferior border of the mandible at the planned implant site (Figure 2B). Adequate mandibular height is essential for both IAN repositioning and implant placement. Severely atrophic posterior mandibles may require preliminary augmentation before or during nerve manipulation [7,8,13]. Cases with a total mandibular height > 10 mm may generally be managed with nerve repositioning alone. In contrast, cases presenting with a total mandibular height ≤ 10 mm may require vertical bone augmentation, which can be performed simultaneously with nerve repositioning [16].

**3.**  **Ridge width**

Because the posterior mandible is subjected to high occlusal loads, restoration typically relies on standard-diameter implants to optimize stress distribution. Consequently, a minimum crestal ridge width of approximately 7.0 mm is generally required to allow implant placement while maintaining an adequate buccal and lingual bone envelope (Figure 2C) [17,18]. Ridges narrower than this are poor candidates for nerve repositioning alone, because IAN lateralization or transposition increases vertical availability but does not correct horizontal ridge deficiency; such cases should be considered for lateral ridge augmentation or expansion [19]. In addition, the mandibular width beneath the IAN canal should be sufficient, commonly >6 mm, to support apical anchorage and facilitate primary stability after nerve mobilization [16,20].

**4.**  **Bone quality**

Bone density may influence both the surgical approach and implant outcomes in IAN repositioning (Figure 2D). Dense cortical bone (D1) increases osteotomy difficulty and the risk of thermal injury to the nerve, thereby favoring piezosurgery over rotary instruments [21,22]. The complexity of nerve exposure is further increased when a thick buccal cortical plate accompanies D1 bone quality, as these conditions complicate window preparation and increase the risk of mechanical and thermal injury to the neurovascular bundle. These factors should be regarded as critical considerations during preoperative planning and case selection [23]. Conversely, D3–D4 bone may compromise primary stability; in such cases, nerve mobilization enables placement of longer implants with bicortical engagement to compensate for reduced bone density [16]. Bone quality was categorized using the conventional D1–D4 scheme based on subjective assessment of cortical and trabecular architecture on CBCT cross-sections. CBCT grayscale values are affected by scanner, field of view, exposure parameters, reconstruction settings, and calibration, and are not directly equivalent to standardized Hounsfield units; D1–D4 assignment on CBCT should therefore be regarded as an approximation rather than a quantitative measurement.

## 5. Prosthodontic Considerations

**5.**  **Interforaminal dentition and technique selection**

Preservation of intact or restorable interforaminal teeth is an important treatment-planning consideration in partially edentulous patients with severe posterior mandibular atrophy. In such patients, IAN repositioning may allow posterior fixed implant rehabilitation while avoiding extraction of existing anterior teeth solely to convert the case to a full-arch tilted-implant concept. This approach is consistent with a conservative prosthodontic philosophy that favors preservation of strategically valuable teeth when they are restorable and periodontally maintainable, and with published clinical experience describing IAN repositioning in partially edentulous mandibles [24].

When IAN repositioning is selected, the presence of interforaminal teeth may also influence technique selection. Because transposition involves anterior relocation of the neurovascular bundle at the mental foramen and interruption of the incisive branch, lateralization is generally preferred when posterior implant placement can be achieved without anterior relocation of the mental foramen, particularly when premolars or anterior teeth are present or restorable [6]. This recommendation should be interpreted as a pragmatic clinical preference based on anatomical considerations and the authors’ clinical experience, rather than as a validated rule.

**6.**  **Crown-to-implant ratio**

The crown-to-implant (C:I) ratio has traditionally been regarded as a biomechanical concern in posterior fixed restorations because increased crown height may lengthen the lever arm and elevate bending moments, particularly under non-axial loading (Figure 2E) [25,26]. However, a recent systematic review suggests that high C:I ratios alone may not significantly increase marginal bone loss or implant failure, particularly when prostheses are splinted [27]. In the context of IAN repositioning, enabling placement of longer implants may still be considered a biomechanical advantage by reducing crown height-related leverage, particularly for non-splinted restorations in high-load posterior regions [41].

**7.**  **Ridge and implant angulation for restoration**

Implant trajectory must align with the planned prosthetic envelope. The angulation of the alveolar ridge relative to the occlusal plane influences the feasibility of ideal implant positioning (Figure 2F). Although no universally validated angular cutoff exists for IAN repositioning, this parameter should be assessed quantitatively on CBCT-based cross-sectional and virtual planning views by measuring the discrepancy between the native ridge axis and the planned prosthetic implant axis. Marked angulation that prevents screw-access emergence within the planned prosthetic envelope, compromises restorative space, or requires excessive buccal/lingual correction should prompt consideration of guided implant placement, adjunctive augmentation, or an alternative treatment strategy. During IAN repositioning, maintaining the native ridge axis and using static surgical guides or navigation systems may help achieve more favorable prosthetically driven implant positioning [23,29,30].

**8.**  **Intermaxillary space**

Intermaxillary space is a key prosthodontic determinant in posterior mandibular rehabilitation. Excessive interarch distance complicates prosthetic design and may require hybrid or overdenture restorations to optimize esthetics and function [28]. Although nerve repositioning increases implant length and bone support, it does not alter the vertical prosthetic dimension; therefore, a comprehensive prosthodontic plan, including occlusal load management and potential splinting, remains essential. In contrast, when intermaxillary space is reduced and residual mandibular height above the IAN canal is limited, vertical augmentation may be prosthetically unfavorable, especially when intact maxillary antagonist teeth are not intended for modification (Figure 2G). In such cases, IAN repositioning may represent a practical alternative for enabling posterior implant placement without altering the opposing dentition.

## 6. Surgical Considerations

**9.**  **Buccal cortical plate thickness**

Buccal cortical plate thickness influences both the design of the lateral access window and the feasibility of replacing it at closure (Figure 2H). A thick, intact cortex allows for the creation of a replaceable bony window that can be repositioned to protect the nerve and help restore ridge contour; however, it also generally requires more demanding osteotomy preparation and may increase the working distance to the IAN, which should be anticipated during window design and instrumentation. In contrast, thin or porous cortices are more prone to fragmentation during osteotomy and may necessitate membrane coverage and/or particulate grafting rather than replacement of the bony window [16,23].

**10.**  **IAN bucco-lingual position**

The bucco-lingual orientation of the mandibular canal varies considerably along the posterior body of the mandible. When the IAN courses close to the buccal cortex, surgical access is generally facilitated, although the risk of cortical perforation and direct nerve injury during window preparation may increase. Conversely, a lingually positioned canal typically requires deeper internal bone removal and meticulous handling of the neurovascular bundle. In cases where the IAN follows a distinctly lingual course (Figure 2I), nerve transposition may be preferable to simple lateralization to avoid excessive tensile stress during mobilization. This scenario often necessitates a more posterior extension of the osteotomy and nerve exposure to permit controlled repositioning and facilitate implant placement in the molar region. CBCT evaluation of the canal’s three-dimensional course is essential to determine the safest window location and the extent of exposure required [8,10].

**11.**  **IAN vertical position**

The vertical depth of the mandibular canal is a key anatomic determinant of both the required osteotomy height and the degree of nerve exposure. When the canal is positioned more superiorly (Figure 2J), a wider unroofing window and more extensive nerve mobilization are typically necessary; conversely, a deeper canal may permit more limited lateralization with reduced exposure. Regardless of canal depth, the anterior loop of the mental nerve should be identified preoperatively on imaging to minimize the risk of postoperative sensory disturbance. In cases of an extremely superficial IAN, in which the bone overlying the nerve is minimal (1–2 mm), access for mobilization can be achieved by removing the thin residual bony layer directly superior to the nerve along the crest (i.e., “Superioralization” of the IAN, a technique described by one of the present authors) [31].

**12.**  **Mandibular Canal diameter**

The diameter of the mandibular canal is a relevant, but often overlooked, parameter in preoperative planning for nerve repositioning. CBCT visualizes the mandibular canal but does not directly image the inferior alveolar nerve. Thus, the canal diameter is used as an indirect anatomical surrogate for the size of the neurovascular bundle. Anatomical studies have reported mean mandibular canal diameters ranging from 2.0 to 2.5 mm in the posterior mandible. A larger canal diameter may reduce the amount of lateral displacement that can be achieved safely and may increase the risk of traction injury during mobilization. Preoperative measurement of canal diameter on CBCT cross-sections is therefore recommended although no clinically validated tensile limit for the inferior alveolar nerve has been established and the measurement cannot define a safe displacement threshold [33].

**13.**  **Bifid canal as a relative contraindication**

IAN repositioning should be regarded as relatively contraindicated in the presence of a bifid mandibular canal because of the increased anatomic complexity and heightened risk of iatrogenic nerve injury. A bifid canal may not be fully appreciated on conventional imaging and may follow unpredictable courses. During lateralization, these additional branches are at greater risk of traction, compression, or transection, potentially leading to neurosensory disturbances [34]. Moreover, inadequate identification of all canal components may result in incomplete nerve mobilization or excessive manipulation. Therefore, when a bifid mandibular canal is detected—particularly on CBCT—alternative treatment strategies, such as short implants, angled implants, or ridge augmentation, should be considered to minimize the risk of permanent nerve damage. The clinical significance of a bifid canal is not uniform and may depend on its type, diameter, origin, and course; the recommendation is therefore relative rather than absolute.

**14.**  **Ridge contour**

The posterior residual ridge frequently presents with a concave contour [38], which, although insufficient for conventional implant placement without nerve manipulation, may still permit simultaneous IAN mobilization and immediate implant placement. For reproducibility, ridge contour should be evaluated on serial CBCT cross-sections by documenting the presence of concavity, knife-edge morphology, undercuts, and the residual bucco-lingual width at the planned implant platform and apical levels. Ridge concavity is a common morphological feature at this stage of atrophy and may compromise ideal implant positioning, soft tissue support, and long-term plaque control if not properly addressed [42].

To optimize ridge morphology and implant stability, adjunctive bone augmentation is often indicated. This may include block grafting or guided bone regeneration (GBR) to increase peri-implant bone volume, cover exposed implant surfaces, and correct ridge concavity. In selected cases, simultaneous vertical augmentation can be performed to level the ridge crest, improve the prosthetic emergence profile, and facilitate oral hygiene after rehabilitation. Adequate ridge correction at this stage plays a critical role in reducing biomechanical overload and enhancing long-term peri-implant tissue stability [39,43].

Irregular or knife-edge crests may impede guide seating and flap closure (Figure 2K). Conservative alveoloplasty can regularize the ridge to facilitate implant alignment and tension-free closure [40]. When ridge reduction is required, the amount of planned alveoloplasty should be measured preoperatively because even minor crestal reduction may further decrease the available vertical height above the IAN.

## 7. Discussion

The management of the severely atrophic posterior mandible remains a clinical challenge that requires integration of anatomical, prosthodontic, and surgical considerations. Although IAN repositioning has been practiced for nearly five decades since Alling’s original description in 1977 [4], treatment planning has largely relied on individual surgeon experience rather than on a standardized framework. The present article proposes a structured, parameter-based clinical decision framework that classifies the key determinants into three clinical domains, aiming to support systematic case selection and technique planning. The contribution of the present work lies in restructuring individual parameters, most of which are already established, into a single multidisciplinary pathway. This framework complements existing classification systems, including the recently proposed Kablan classification [16], which stratifies IAN repositioning procedures according to mandibular height, width, angulation, and residual bone height above the IAN. Thus, the present framework offers a broader preoperative planning purpose. Rather than replacing this anatomical classification, it expands the decision-making process by integrating prosthodontic and surgical risk parameters, including interforaminal dentition, crown-to-implant ratio, intermaxillary space, canal position, mandibular canal diameter, bifid canal anatomy, and ridge contour. In this way, the proposed framework translates anatomical classification into a multidisciplinary treatment-selection pathway.

The bone-related parameters outlined in the present framework align with and extend the anatomical criteria described in existing classification systems. The Kablan classification stratifies cases into four categories based on mandibular height, width, angulation, and bone height above the IAN, with surgical staging defined accordingly [16]. The present framework incorporates these dimensional criteria while adding bone density as an independent factor influencing both surgical approach and prognosis. This distinction is clinically relevant because two mandibles with identical dimensions but different bone densities may require fundamentally different osteotomy techniques and may carry different risks of complications. The inclusion of bone quality also highlights the role of piezosurgery as a risk-reduction strategy in dense cortical bone, a recommendation supported by in vitro evidence demonstrating less nerve damage with piezoelectric instruments than with conventional rotary burs [21] and by clinical data showing faster neurosensory recovery [44]. Furthermore, by establishing minimum thresholds for mandibular height and width below which adjunctive augmentation is required, the framework may help clinicians avoid performing nerve repositioning in cases where the residual bone volume is insufficient to support predictable implant anchorage, even with longer fixtures.

The inclusion of prosthodontic parameters as an independent domain distinguishes the present framework from purely anatomical classification systems. Although a mandible may satisfy all bone-related criteria for IAN repositioning, the planned restoration may render the procedure either unnecessary or insufficient. For example, when excessive intermaxillary space necessitates a hybrid or overdenture prosthesis, the biomechanical advantage of longer implants may be offset by unfavorable lever-arm mechanics, making alternative approaches equally appropriate. This perspective is consistent with emerging evidence indicating that the crown-to-implant ratio, long regarded as a critical biomechanical determinant, may be less influential on marginal bone loss than previously assumed, particularly when prostheses are splinted [27]. In severely resorbed cases, however, IAN repositioning may represent the only viable route to a fixed implant-supported prosthesis [20,24,45], offering practical advantages by permitting immediate implant placement and reducing overall rehabilitation time compared with staged grafting protocols [2,46].

Surgically, the choice between lateralization and transposition is guided by anatomical constraints and risk stratification. Lateralization, which displaces the nerve posterior to the mental foramen without transecting the incisive branch, is generally preferred because of its lower complication profile [6]. Transposition, which involves repositioning the mental foramen and sectioning the incisive nerve, is reserved for anatomically demanding cases in which implant placement must extend into the foramen region, since only anterior release of the neurovascular bundle creates access to this region [6,47]. A distinctly lingual canal course is a contributing rather than a determining factor: it increases the depth of dissection and the degree of mobilization required, and may favor transposition when combined with a need for posterior extension of the osteotomy. However, it does not by itself justify sectioning the incisive branch. No validated threshold defines the point at which the required degree of nerve mobilization becomes excessive for lateralization; this remains an intraoperative judgment based on the tension observed during retraction and on the length of nerve exposed. Both procedures should be reconsidered in favor of a lower-risk alternative when a bifid canal is identified, when residual bone dimensions remain inadequate for implant anchorage even after repositioning, when the planned restoration is not prosthodontically feasible, or when patient- or operator-related factors increase the risk of an adverse neurosensory outcome.

The difference in neurosensory risk between the two techniques should be interpreted with care. Systematic analyses have documented temporary neurosensory disturbance in approximately 95.9% of patients after lateralization cases initially, decreasing to 3.4% at final follow-up, compared with 58.9% initially and 22.1% persisting after transposition [3]. These figures should not be interpreted as a direct comparison. They derive from separate and largely non-overlapping sets of primary studies, seven reporting lateralization, fifteen reporting transposition, and two reporting both, with follow-up ranging from 6 to 49.1 months, and the included studies were not standardized with respect to the timing or method of neurosensory assessment. The contrast between a higher early rate after lateralization and a higher persistent rate after transposition therefore reflects differences between study populations and assessment protocols as much as any true difference between the techniques. Despite these complication rates, implant survival following IAN repositioning remains consistently high. Martinez-Rodriguez et al. reported 98.6% implant survival at 5-year follow-up after lateralization, with nearly all initial neurosensory disturbances resolving by 18 months [48]. Gasparini et al. also confirmed favorable long-term outcomes in a cohort of 35 patients followed for up to 101 months after transposition [49]. These two cohorts likewise differed in design, follow-up duration, and outcome definition, and are not directly comparable.

The present framework incorporates specific surgical parameters that influence both technique selection and complication risk. Buccal cortical plate thickness determines the feasibility of creating a replaceable bony window versus relying on membrane coverage. The bucco-lingual position of the canal dictates the depth of internal bone removal required, with lingually positioned canals demanding more extensive dissection. Canal diameter, an underinvestigated parameter, also warrants preoperative assessment: mean canal diameters of 2.0–2.5 mm have been reported in the posterior mandible [33], and a larger neurovascular bundle may be more susceptible to traction-related neuropraxia and axonotmesis during mobilization [32]. Furthermore, the presence of a bifid mandibular canal should be regarded as a relative contraindication to IAN repositioning. CBCT-based anatomical studies have shown that bifid canals are far more prevalent than previously recognized on conventional imaging, with detection rates ranging from 15.6% to 65% depending on the classification criteria used [35]. Notably, the diameter of the accessory canal has been shown to reach 50% or more of the main canal in nearly half of cases [36], and histological investigation has confirmed that these accessory canals contain functional nerve bundles and blood vessels rather than representing mere trabecular artifact [37]. The unpredictable course of these accessory neurovascular branches increases the risk of incomplete mobilization or iatrogenic injury, as unidentified branches may be severed or subjected to uncontrolled traction during nerve manipulation.

The proposed clinical decision pathway integrates the parameters described above into a sequential workflow that guides the clinician from initial indication assessment to technique selection. The pathway begins with the primary gating criterion of residual bone height above the IAN (<6 mm) and then systematically evaluates global mandibular volume, ridge width, and bone quality to determine whether nerve repositioning alone may be sufficient or whether adjunctive augmentation should be considered. Prosthodontic parameters are assessed in parallel to determine whether the planned restoration is compatible with the achievable implant position and angulation. Finally, surgical parameters, including cortical thickness, canal position, and canal anatomy, inform the choice between lateralization and transposition, as well as the specific osteotomy approach. When more than one implant site is planned within the same segment, each parameter should be measured at every planned site rather than at a single representative location, since anatomical values commonly vary along the segment. Because the pathway determines how the segment as a whole will be treated, the most unfavorable planned site should govern the decision nodes: if any planned site fails a given criterion, the corresponding branch is followed. Where an unfavorable value is confined to a single site, a site-specific measure, such as localized augmentation, a reduced-diameter implant, or omission of that site from the restorative plan, may be preferable to applying a more invasive pathway across the whole segment. The choice between these options should be made explicitly and recorded as part of the plan rather than left to default. This structured sequence offers several important insights. First, the present framework explicitly incorporates prosthodontic planning as an independent domain, recognizing that anatomical suitability for nerve repositioning does not automatically translate into prosthetic suitability. Second, the flowchart includes decision nodes at which alternative treatments, such as short implants, ridge splitting, or vertical augmentation, are positioned as viable treatment pathways rather than fallback options, thereby helping clinicians consider lower-risk alternatives when appropriate. Comparison between IAN repositioning and its alternatives should not rest on available bone height alone. Short implants avoid neurosensory risk entirely and require shorter treatment times, and the evidence that unfavorable crown-to-implant ratios compromise survival is not consistent. Vertical augmentation restores ridge height but entails staged surgery, graft-related morbidity, and variable resorption. Ridge splitting and angled implants address specific deficiencies at lower neurological risk. IAN repositioning offers single-stage fixed rehabilitation but carries the highest neurosensory morbidity of the available options. Treatment duration, staging, prosthetic design, maintenance requirements, patient preference, and the patient’s willingness to accept temporary or permanent altered sensation should all guide the choice, and the biomechanical advantage of longer implants should not by itself justify a procedure with this risk profile.

The present framework should be interpreted as a conceptual planning aid rather than as a validated clinical decision tool. It has not yet been validated in a prospective or retrospective patient cohort, and this represents an important limitation. In addition, the framework was developed from a focused, selective appraisal of the literature combined with the authors’ clinical experience, rather than from a systematic review, formal guideline methodology, or expert consensus process, and no formal risk-of-bias or certainty-of-evidence assessment was performed. Several components of the framework draw on the authors’ own prior work, and the relative contribution of the previously published classification and of the present framework is set out above. None of the proposed thresholds has been validated against clinical outcomes in the context of IAN repositioning, and the binary form in which some are presented in Figure 1 aids legibility of the decision pathway rather than reflecting the strength of the underlying evidence.

Future validation should include multicenter retrospective application of the proposed decision pathway to previously treated patients, followed by prospective assessment in consecutive patients undergoing treatment planning for posterior mandibular atrophy. Relevant validation outcomes should include agreement between clinicians in applying the framework, concordance between the proposed pathway and the final treatment selected, neurosensory outcomes, implant survival, need for adjunctive augmentation, prosthetic feasibility, and patient-reported outcomes. Consensus-based refinement by clinicians experienced in implant rehabilitation of the atrophic posterior mandible may further help determine whether specific thresholds or decision nodes require modification before broader clinical implementation. The current framework addresses local anatomical, prosthodontic, and surgical planning, and does not incorporate patient-related factors such as existing sensory disturbance, diabetes, smoking, impaired healing, antiplatelet or anticoagulant therapy, previous mandibular surgery, parafunction, oral hygiene, or the patient’s acceptance of altered sensation, nor operator-related factors such as experience in nerve mobilization and in the management of postoperative neurosensory complications. A procedure may be anatomically feasible yet inappropriate for a given patient or in a given centre. The framework therefore cannot independently determine overall patient suitability and must be applied alongside a full clinical assessment. Preliminary retrospective validation was not undertaken for the present article because the framework was formulated during its preparation, and a retrospective application to cases treated before the pathway existed would test only concordance with the authors’ own prior practice rather than the reproducibility or clinical utility of the framework.

## 8. Conclusions

In conclusion, this article presents a literature-informed, expert-derived clinical decision framework that integrates bone-related, prosthodontic, and surgical considerations into a single preoperative planning pathway for IAN repositioning. The novelty of this framework lies in its multidisciplinary structure, which extends beyond anatomical classification alone and incorporates restorative feasibility, surgical risk factors, and alternative treatment pathways. The proposed framework may help structure multidisciplinary assessment and discussion of when inferior alveolar nerve lateralization or transposition may be considered and when adjunctive or alternative approaches may be more appropriate. However, it should not be regarded as a validated clinical decision tool. Future retrospective and prospective validation is required to assess its reproducibility, clinical utility, and potential impact on treatment selection and outcomes. Until such validation is available, the framework should not independently determine treatment, replace individualized clinical judgment, or be taken to imply improved clinical outcomes.

## Data Availability

The original contributions presented in this study are included in the article. Further inquiries can be directed to the corresponding author.
